# 

**Published:** 1892-02

**Authors:** 


### CONTENTS FOR FEBRUARY, 1892.

ORIGINAL COMMUNICATIONS.

Transverse Presentations.—History of a Case with a New Method of Treatment. By EUGENE EVERETT BARNUM, A. M., M. D. .......... 386
Clinical Contributions to Brain Surgery. By JOHN B. ROBERTS, M. D .......... 389

SOCIETY PROCEEDINGS.

Transactions of the Fourth Annual Meeting of the American Association of Obstetricians and Gynecologists .......... 407
The Medical Society of the County of Erie .......... 424
Harlem Medical Association .......... 431

EDITORIAL.

The Physician in Politics .......... 439
Editorial Melange .......... 440
The International Executive Committee of the Pan-American Medical Congress .......... 444
The Pan-American Medical Congress in the United States of Colombia .......... 445

BOOK REVIEWS.

Regional Anatomy in its Relation to Medicine and Surgery .......... 446

